# Non-invasive respiratory support in the management of COVID-19: a synthesis of systematic reviews

**DOI:** 10.12968/bjca.2022.0060

**Published:** 2023-01-19

**Authors:** James Edward Hill, Abdelaal Hebatalla, Oliver Hamer, Katalin Ujhelyi Gomez, Joanna Harrison, Thomas Bongers

**Affiliations:** 1 University of Central Lancashire, Colne, UK; 2 Blackpool Teaching Hospitals NHS Foundation Trust, Blackpool, UK; 3 University of Liverpool, Liverpool, UK

## Introduction

In December 2019, an outbreak of acute respiratory infections spread through Wuhan, China (1). These infections were discovered to be a novel coronavirus (severe acute respiratory syndrome coronavirus-2 or SARS-CoV-2), termed COVID-19 by the World Health Organisation (1). The COVID-19 outbreak is believed to have originated via a zoonotic (vertebrate animals) spread which later transmitted to humans (2). The rapid spread of the virus was declared a global pandemic in March 2020, as cases grew 13-fold within a brief period (3). COVID-19 is primarily transmitted from human to human via respiratory droplets (spread by coughs or sneezes) and fomites (materials such as clothes, furniture, etc.) which are likely to carry infection used by, or on those infected (1, 3).

By late 2021, there were over 280 million confirmed cases and over 5 million deaths globally (4, 5). In the UK, there were over 22 million confirmed COVID-19 cases with over 800,000 patients admitted into hospital and a number of these requiring mechanical ventilation (6). Patients with COVID-19 often present with a dry cough, fever, fatigue, breathlessness, malaise and some present with gastrointestinal symptoms (7). COVID-19 has an impact on multiple organs, but respiratory failure is considered the major cause of COVID-19 mortality (8). Mortality is likely to be caused by the strong negative effect COVID-19 has on the respiratory system and its association with acute respiratory distress syndrome (ARDS) (9).

Hospitalised patients with COVID-19 often require oxygen because of the increased risk of hypoxia (10). One of the main treatment goals is to avoid mechanical ventilation, where possible, (10). Non-invasive respiratory support (NIRS) such as high flow nasal oxygen (HFNO), bi-level positive airway pressure (BiPAP) and continuous positive airway pressure (CPAP), appear to be clinical alternatives (favoured by healthcare professionals) (10). CPAP may be the most appropriate NIRS treatment for COVID-19 as it delivers a constant flow of oxygen at a continuous pressure during both phases of breathing to prevent the collapse of the alveoli, increasing the lung volume, managing respiratory failure and improving gaseous exchange (11). However, further research is needed to establish its effectiveness as a treatment for COVID-19 within a diverse population.

## Aim of commentary

This commentary aims to critically appraise three reviews concerning the use of NIRS in patients with COVID-19 and expand upon the findings in regard to clinical practice.

## Methods and Quality of the Reviews

The core inclusion criteria were similar within all three systematic reviews (12–14). These were that only studies which included patients with COVID-19 who had undergone non-invasive respiratory support (e.g., BiPAP, HFNO, or CPAP) were included in the three reviews (12–14). Two of the reviews included Randomised Controlled Trials (RCTs) and observational studies, whilst one review focused only on international and national guidelines (12). Mortality was the primary outcome reported in all three reviews (see Table 1 for full PICOS).

All three reviews were deemed to be methodologically robust. Each review clearly stated the research question, described appropriate inclusion criteria, outlined the search strategy, conducted critical appraisals with two or more reviewers, and combined study data using appropriate analysis methods (see Table 2 for full critical appraisal and corresponding methods used for each review). The only areas of concern related to the assessment of publication bias and the data extraction methods. Two reviews did not investigate or report on publication bias (12, 13). Furthermore, none of the reviews provided adequate detail to determine if more than one author had verified the data extraction.

## Results

### Radovanovic et al (2021)

After duplicate removal, a total of 1,150 records were identified of which 23 studies were included. The studies included a total of 4,776 patients with COVID-19. Of the studies, more than 74% were conducted in Europe (n=17), 13% in China (n=3) and one study each in Pakistan, Egypt and Russia. Twenty-one studies were retrospective observational studies from a single site and two studies were prospective studies.

About half of all patients in the included studies received NIRS of which 49% received CPAP, 46% received BiPAP and 4% received CPAP and/or BiPAP. From the 13 studies which reported the pressure of oxygen/fraction of inspired oxygen (PaO^2^/FiO^2^) before starting CPAP and/or BiPAP, the mean PaO^2^/FiO^2^ was <200 mmHg (moderate-to-severe respiratory failure) (15). From the eight studies which reported positive end expiratory pressure, the mean pressure was 10 cmH_2_O. All studies were critically appraised to be at a quality standard of at 3/3 stars using the Newcastle Ottawa scale for both population selection and outcome. Due to all studies being observational, the criteria for comparability were not applicable.

Failure of NIRS (due to decreased level of consciousness, exhaustion, refractory hypoxemia, sepsis, hemodynamic instability) occurred on average 56% of the time (in all studies). The use of CPAP resulted in intubation (26%) and mortality 22% of the time on average (17 studies). The use of BiPAP resulted in intubation (24%) and mortality 25% of the time (13 studies). The length of stay within included studies ranged from 6.2 to 21 days. None of the studies included a control group because most were retrospective, and all were non-randomized studies. Notably, CPAP/BiPAP was applied as a standard of care procedure in patients that required it according to pre-defined criteria (e.g. persistent hypoxemia despite oxygen supplementation and/or respiratory distress).

### Weerakkody et al (2021)

Eighty-four articles (78 studies) were included in the review with a total of 13,931 patients with COVID-19 pneumonia. Of these, most papers were from Western Europe (62 studies), nine from China and seven from the USA. The majority were of observational design of which 61 studies were retrospective, 22 were prospective, and two were RCTs. Outcomes were specifically reported for CPAP in 29 studies, HFNO in 23 studies, two modalities in 17 studies and BiPAP in six studies. The specific modalities used in the remaining three studies were not specified. From the 16 studies which reported positive end expiratory pressure, the median pressure was 10 cmH_2_O (IQR 7·5–11·0). The observational studies were too heterogenous to conclude the superiority of one technique compared to another. The review did not clarify how the quality of evidence was evaluated/appraised.

The overall survival in patients receiving NIRS was 66% with survival reported from 7 to 60 days (85 studies). The survival rate for 60 days was slightly less at 65% (59 studies). The review reported the outcome into three categories of patients; patients with NIRS as the ceiling of treatment, patients for full escalation to invasive ventilation, and patients where escalation was not specified.

In patients with NIRS as ceiling of treatment, the median duration of NIRS treatment was reported in five studies, with a median survival rate of 29% (IQR 15-45) [survival cut-off ranging from 28 to 60 days or discharge]. Duration of treatment was shorter for those in whom NIRS was deemed to be a failure. Full escalation progression to invasive mechanical ventilation occurred in 37% of patients who initially started on NIRS (40 studies). Overall survival using each study end value was 78%, with median survival almost the same with all NIRS modalities [79% (IQR 73-89), 83%, 76% for CPAP, HFNO, BiPAP respectively]. Median duration of NIRS treatment was longer in patients for whom NIRS was a success. In patients where escalation was not specified, progression to invasive ventilation occurred in patients initially started on HFNO in 42% (IQR 30–54, 12 studies), CPAP in 29% (20–36, 8 studies) and BiPAP in 23% (16–38, 3 studies) of the time when used as a sole modality, and 35% (IQR 23–44) of the time in patients who received CPAP, BiPAP or HFNO (21 studies). Overall survival was 64.1% and median survival was almost similar with all individual modalities of 70% (IQR 53-81), 69% (IQR 63-87), 62% (IQR 28-75) for HFNO, CPAP and BiPAP respectively. Reported median duration of NIRS treatment was longer in patients for whom intervention was a success.

In one RCT, intubation occurred statistically significantly less in patients given BiPAP (30%) compared to HFNO (51%). However, there was no difference between the groups’ mortality. In a further RCT, intubation or mortality (30 days) was statistically significantly less in patients given CPAP (36%) than oxygen therapy (44%). There was also no statistically significant difference between oxygen therapy and HFNO.

Several moderating factors for increased risk of NIRS failure were identified. These were older age, male sex, comorbidities, severity of illness on admission, higher respiratory rate, oxygenation on admission, ROX index (respiratory rate oxygenation: defined as the ratio of oxygen saturation as measured by pulse oximetry (SpO2)/FiO2 to respiratory rate (RR), change in ROX index in response to NIRS and higher inflammatory markers. In associations between time to invasive ventilation and mortality, there was a wide range of conclusions from different studies. Some studies reported higher mortality with earlier intubation, while others reported increased mortality risk with delayed intubation.

### Wang et al (2021)

Screening of 108 articles identified 26 guidelines that were selected for evaluation. The guideline recommendations provided guidance for four clinical practice related themes: safety issues (n=37), optimization of NIRS installation (n=20), indications for the use of NIRS (n=49), and modes and parameter settings (n=22).

#### Safety issues

Several recommendations were made regarding safety issues of NIRS. Firstly, 13 recommendations described concerns that NIRS generates aerosol and therefore patients should be situated in a single isolated room, a negative-pressure ward, or a ward dedicated to NIRS treatment. Medical staff also need to be aware of the procedures associated with aerosol management. Secondly, five recommendations stated that medical staff should wear full personal protection equipment when treating patients with NIRS (e.g. eye protection, N95 or higher respirators, gloves, and long-sleeved gowns). The quality of evidence and strength of these recommendations was ungraded and weak.

#### Optimization of NIRS installation

Recommendations for the optimization of NIRS installation focused on the use of helmet NIRS and antimicrobial filters. Sixteen recommendations suggested that helmet NIRS should be a first-choice mode because it is more tolerable and reduces room contamination which may increase the safety of other healthcare staff. If a helmet cannot be used, the use of masks combined with a double circuit expiratory valve is recommended. Another key guideline related to the use of antimicrobial and antiviral filters, which, according to four recommendations, should be installed to limit exhaled air dispersion into the setting. The quality of evidence and strength of these recommendations could not be graded.

#### Indications in the use of NIRS

Recommendations for indications in the use of NIRS set out guidance on patient monitoring, use with respiratory diseases, hemodynamic instability, multi-organ failure and mental health conditions. Fifteen guidelines proposed that patients undergoing NIRS should be monitored closely for at least one hour and up to four hours following ventilation. However, the strength of evidence for these recommendations was weak and of low quality. A further 31 recommendations suggested that patients with worsening respiratory status, mental health concerns, hemodynamic instability or multi-organ failure, should not receive NIRS if other options are available (e.g. invasive ventilation/early endotracheal intubation). The strength of this evidence ranged from strong to weak, dependant on the evaluation of the individual publication. The evidence that could be graded was deemed to be of moderate quality.

#### Modes and parameter settings

Five recommendations for modes and parameter settings described how CPAP and BiPAP may be considered for specific patient groups. These modes should only be considered in patient groups with type 2 respiratory failure (e.g. chronic obstructive pulmonary disease) and should not be used in individuals who are not spontaneously breathing. However, these recommendations could not be graded for strength or quality. A further six recommendations related to parameter setting stated that low-flow CPAP was suitable for patients with a lower oxygen requirement (fraction of inspired oxygen, FiO^2^ < 0.4). In well-orientated patients, CPAP flow may be set to 10–15 ^x^cm/H_2_O with FiO^2^ at 0.6 – 1.0 if no side effects are observed. Although these recommendations were provided by several guidelines included in the review, the quality and strength of evidence could not be graded. In patients where escalation is required, CPAP pressures may be increased to 15–20 ^x^cm/H_2_O. The final recommendation related to the modes and parameter settings describing S_p_O^2^ targets for different patient populations. Eleven recommendations stated that patient blood oxygen saturation (SpO2) targets should be above 90% but no higher than 96%. However, S_P_O^2^ targets for patients with evidence of acute or chronic type 2 respiratory failure should be between 88– 92%. The strength of this recommendation was strong and of moderate quality.

Overall, the recommendations synthesised in the review were primarily based on clinical expertise of viral pneumonia conditions or the conclusions of observational studies. These recommendations must be interpreted with caution as many were poor quality, with little stakeholder involvement and a lack of rigorous development.

### Commentary

Critical appraisal using the Joanna Briggs Checklist for Systematic Reviews determined that all three reviews were methodologically robust (16). However, there were some concerns that data extraction was not conducted by two authors independently, and that publication bias was not assessed in the reviews (See Table 2 for critical appraisal and corresponding methods for all reviews) (12–14). The systematic review by Weerakkody et al (2021) did not fully describe the method of quality assessment. However, there was an inclusion criterion for exclusion of poor-quality case series studies but there was no description as to how this judgement was undertaken. Additionally, Radovanovic et al. (2021) did not stipulate the exact methods of synthesis. This lack of clarity makes it difficult to assess the accuracy of the mean estimates presented within the review and should therefore be viewed with some caution. The systematic review by Wang et al. (2021) provided an accurate and comprehensive synthesis of the available studies that addressed the question of interest.

Based on retrospective evidence, NIRS is currently used to treat patients with COVID-19 (13, 14). However, failure of NIRS is high among patients, occurring in more than half of those treated (14). There appears to be little difference in success of alternate modes of NIRS, with CPAP and BiPAP showing similar rates of failure with patients with COVID-19 (13, 14). The use of CPAP resulted in approximately a quarter of patients escalated to intubation and death occurring on average in one in five patients. The use of BiPAP led to similar results in escalations to intubation and patient death in approximately a quarter of all patients (14). Subsequently, as identified within a large number of guidelines, it is essential to undertake regular close (1 to 4 hours) monitoring when patients are placed on NIRS (12). As part of this monitoring process, it is important to note that patients who are older, male sex, severely ill on admission, need oxygenation on admission, have a higher respiratory rate, higher inflammatory markers and have a comorbidity are at a higher risk of NIRS failure (13). During this monitoring process, a change in ROX index (ratio of oxygen saturation as measured by pulse oximetry/FIO2 to respiratory rate) may provide an early warning regarding possible risk of NIRS failure (17).

In instances were NIRS may be a suitable treatment option, clinicians should give priority to BiPAP for patient groups with type 2 respiratory failure (e.g. chronic obstructive pulmonary disease) with target oxygen saturation of 88-92% (12). While in patients with type 1 respiratory failure recommended target oxygen saturation should be above 90% not exceeding 96% (13) and recommended/advised starting mean pressure when using CPAP is 10 cm H2O (13, 15). When assessing the appropriateness of NIRS for patients with either worsening respiratory status, mental health concerns, hemodynamic instability or multi-organ failure, alternative methods may be preferable (e.g. invasive ventilation/early endotracheal intubation) (12). Wherever possible, helmet NIRS should be considered. Alternatively, masks combined with a double circuit expiratory valve are recommended (12).

Although the effectiveness of NIRS is somewhat unclear in patients with COVID-19, evidence suggests that it is effective in adult intensive care patients and in patients with severe community-acquired pneumonia (18, 19). In these patient populations, clinical outcomes such as risk of death and length of hospital stay can be improved by employing NIRS as an alternative to standard oxygen therapy (18, 19).

Due to high incidence rates of disease worldwide, there has been a growing demand for mechanical ventilation among healthcare services (20). Consequently, there has been a reduction in resources needed to safely deliver invasive ventilation, including mechanical ventilators and intravenous sedation (21). Due to these shortages, there is a need for alternative treatment options, such as NIRS, to treat COVID-19 related respiratory failure. NIRS could be an effective treatment option when utilized in the appropriate setting, either as a preventive therapy or as a rescue in patients with a therapeutic ceiling (22). However, further research is needed to evaluate factors that influence successful application and initiation of NIRS in patients with COVID-19. Due to the dearth of robust evidence (lack of RCTs), the effectiveness of NIRS should also be explored in patients outside of ICU with other respiratory conditions (e.g. pneumonia) that present similar severity of symptoms. Further high-quality RCTs are needed to provide more reliable recommendations in terms of NIRS use in COVID-19 (considering vaccination programmes that lower the likelihood of COVID-19 related hospitalization and death), or non-COVID-19 related respiratory failure. New evidence has the potential to widen the scope of NIRS implementation and use in comparison to other available treatment modalities. Additionally, research is needed to establish the degree of NIRS related complications including a higher risk of healthcare exposure to the virus.

## Figures and Tables

**Table 1:** PICOS Characteristics

	Study		
PICOS	Radovanovic et al (2021)	Weerakkody et al (2021)	Wang et al (2021)
**P**opulation	Patients with COVID-19 (>18 years)	Patients with COVID-19 pneumonia	Patients with COVID-19
**I**ntervention	Non-invasive respiratory support (CPAP or BiPAP)	Non-invasive respiratory support (CPAP, BiPAP or HFNO)	Non-invasive respiratory support (CPAP or BiPAP)
**C**ontrol	N/R	N/R	N/R
**O**utcomes	Mortality and endotracheal intubation	Survival (cut-off ranging from 28 to 60 days or hospital discharge), escalation to invasive mechanical ventilation, mortality	N/R
**S**tudy type	23 studies, most retrospective and single centre studies, 2 prospective observational design, no RCTs.	2 RCTs and 83 observational studies	Guidelines developed by international or national health organizations or medical societies.
**A**dditional inclusion criteria	N/R	Literature searches were conducted on prespecified inclusion criteria. One large RCT was published as a pre-print after the original search, and included due to its size	Included guidelines from international or national health organizations or medical societies but excluded those not in English

Key N/R- Not reported, CPAP- Continuous positive airway pressure, BiPAP - Bilevel Positive Airway Pressure, HFNO - high flow nasal oxygen, RCTs random controlled trials.

**Table 2:** Critical appraisal (Modified JBI critical appraisal tool) of included reviews

Criteria	Radovanovic et al (2021)	Weerakkody et al (2021)	Wang et al (2021)
1. Is the review question clearly and explicitly stated?	Yes - the review analysed the outcomes such as failure of non-invasive respiratory support in terms of need for endotracheal intubation and mortality in and outside Intensive Care Units (ICU).	Yes - the review provided an overview about outcomes in patients with COVID-19 who received one or more NIRS modalities, examining duration of use, outcomes, predictors of success or failure.	Yes – the review addresses the issues that arise in developing guidelines for a pandemic and enhances clinicians’ understanding of the use of NIRS when facing COVID-19.
2. Were the inclusion criteria appropriate for the review question?	Yes - see Table 1.	Yes – see Table 1.	Yes - see Table 1.
3. Was the search strategy appropriate?	Yes - the search had appropriate terms.	Yes – the search had appropriate terms, including acute respiratory distress syndrome, or acute respiratory failure to improve scope and relevance.	Yes - the search had appropriate terms and domains.
4. Were the sources and resources used to search for studies adequate?	Yes - Multi database search was undertaken including Medline and Embase; in addition to revision of literature. Date limit between December 2019 and November 2020.	Yes - searched PubMed, Embase, ScienceDirect, Scopus, Google Scholar, and medRxiv for relevant studies published in English from January 2020 to June 2021, in addition to literature search on pre-specified inclusion criteria.	Yes - included databases such as PubMed, Web of Science, and Cochrane Library, in addition to websites of international guidelines. Reference lists of included studies were also searched up to June 2020.
5. Were the criteria for appraising studies appropriate?	Yes - risk of bias and study quality were assessed by means of the Newcastle-Ottawa Quality Assessment Scale (NOS).	Unclear.	Yes - the study used the Appraisal of Guidelines for Research & Evaluation Instrument (AGREE II)
6. Was critical appraisal conducted by two or more reviewers independently?	Yes - the critical appraisal of included studies was independently conducted by two authors with arbitration by a third reviewer if consensus was unable to be achieved by discussion.	Unclear.	Yes - four qualified appraisers had been trained through online practice grading and pre-grading to appraise the studies.
7. Were there methods to minimize errors in data extraction?	Unclear.	Unclear.	Unclear – it is unclear if more than one researcher verified the data extraction.
8. Were the methods used to combine studies appropriate?	No - The methods of synthesis were unclear.	Yes - the review divided the studies according to the pre-decided escalation plan for the patients, comparing the outcomes, providing a supplementary table of predictors of failure in each study.	Yes - the review narratively discussed the studies with some numeration analysis to support recommendations.
9. Was the likelihood of publication bias assessed?	No – no formal method of publication bias was assessed however it is mentioned as a possible point of bias of the methods used.	No - the study did not report on publication bias.	Not applicable
Total criteria achieved	7/9	7/9	7/8

## Commentary on

➢ Wang Z, Wang Y, Yang Z, Wu H, Liang J, Liang H, Lin H, Chen R, Ou Y, Wang F, Wang Y, Wang Y, Luo W, Li N, Li Z, Xie J, Jiang M, Li S. The use of non-invasive ventilation in COVID-19: A systematic review. Int J Infect Dis. 2021 May;106:254-261. doi: 10.1016/j.ijid.2021.03.078. Epub 2021 Mar 30. PMID: 33798720; PMCID: PMC8008782.
➢ Weerakkody S, Arina P, Glenister J, Cottrell S, Boscaini-Gilroy G, Singer M, Montgomery HE. Non-invasive respiratory support in the management of acute COVID-19 pneumonia: considerations for clinical practice and priorities for research. Lancet Respir Med. 2022 Feb;10(2):199-213. doi: 10.1016/S2213-2600(21)00414-8. Epub 2021 Nov 9. Erratum in: Lancet Respir Med. 2021 Dec;9(12):e114. PMID: 34767767; PMCID: PMC8577844.
➢ Radovanovic D, Coppola S, Franceschi E, Gervasoni F, Duscio E, Chiumello DA, Santus P. Mortality and clinical outcomes in patients with COVID-19 pneumonia treated with non-invasive respiratory support: A rapid review. J Crit Care. 2021 Oct;65:1-8. doi: 10.1016/j.jcrc.2021.05.007. Epub 2021 May 21. PMID: 34052780; PMCID: PMC8137355.

## Key Points

- There was little difference in success of alternate modes of Non-invasive Respiratory Support (NIRS) with Continuous Positive Airway Pressure (CPAP), High-Flow Nasal Oxygen (HFNO) and Bilevel Positive Airway Pressure (BiPAP) showing similar rates of failure with patients with COVID-19.
- Patients with COVID-19 placed on NIRS should be monitored closely (1 to 4 hours).
- Research is needed to establish whether NIRS is superior to conventional oxygen therapy with regards to mortality and identify the burden of NIRS therapy compared to conventional oxygen therapy.
- Further research is needed to clarify the benefits and risks related to the use of NIRS in patients with COVID-19 compared to oxygen therapy.

## CPD reflective questions

- When determining whether to use NIRS in a patient with COVID-19, what are the key factors that need to be considered?
- What are the key benefits of using NIRS (e.g. CPAP) versus mechanical ventilation in patients with COVID-19?
- What are the limitations and strengths of the systematic reviews?
